# Selinexor plus tislelizumab in patients with relapsed/refractory natural killer/T-cell lymphoma after failure of PD-1 blockade: the phase 1b TOUCH trial

**DOI:** 10.1093/oncolo/oyag266

**Published:** 2026-07-11

**Authors:** Chuanxu Liu, Dahui Li, Yan Gao, Wenhao Zhang, Zhi Guo, Jun Zhao, Huiqiang Huang, Rong Tao

**Affiliations:** Department of Lymphoma and Medical Oncology, Fudan University Shanghai Cancer Center, Shanghai, 200032, China; Research Center for Lymphoma, Fudan University, Shanghai, 200032, China; Department of Lymphoma and Medical Oncology, Fudan University Shanghai Cancer Center, Shanghai, 200032, China; Research Center for Lymphoma, Fudan University, Shanghai, 200032, China; Department of Medical Oncology, Sun Yat-Sen University Cancer Center, Guangzhou, 510060, China; Department of Lymphoma and Medical Oncology, Fudan University Shanghai Cancer Center, Shanghai, 200032, China; Research Center for Lymphoma, Fudan University, Shanghai, 200032, China; Antengene Therapeutics Ltd, Shanghai, 200051, China; Antengene Therapeutics Ltd, Shanghai, 200051, China; Department of Medical Oncology, Sun Yat-Sen University Cancer Center, Guangzhou, 510060, China; Department of Lymphoma and Medical Oncology, Fudan University Shanghai Cancer Center, Shanghai, 200032, China; Research Center for Lymphoma, Fudan University, Shanghai, 200032, China

**Keywords:** extranodal NK/T-cell lymphoma, selinexor, tislelizumab, XPO1 inhibition, PD-1 blockade resistance

## Abstract

**Background:**

Relapsed or refractory extranodal natural killer/T-cell lymphoma (R/R NKTCL) remains a highly lethal disease, particularly after failure of PD-1 blockade–based therapy. Preclinical data suggest that inhibition of exportin-1 (XPO1) may enhance antitumor immunity and synergize with PD-1 blockade.

**Patients and Methods:**

We conducted a multicenter, open-label phase 1b study (TOUCH, Arm C) evaluating selinexor plus tislelizumab in patients with R/R NKTCL previously treated with L-asparaginase–containing regimens. A standard 3 + 3 dose-escalation design was followed by dose expansion.

**Results:**

Seventeen patients were enrolled; 16 had prior checkpoint inhibitor (CPI) exposure and comprised the efficacy population. No dose-limiting toxicities were observed. Grade ≥3 treatment-emergent adverse events occurred in 52.9% of patients; hematologic toxicities were the most common. Among patients with prior CPI exposure, the overall response rate was 75.0% (12/16), including complete responses in 43.8% (7/16). Responses were observed in patients with primary CPI-refractory disease. With a median follow-up of 21.7 months, median progression-free survival was 6.1 months (95% CI, 2.9–not estimable), and the 2-year progression-free survival rate was 37.5%. Median overall survival was not reached; the 2-year overall survival rate was 73.4%.

**Conclusion:**

Selinexor plus tislelizumab demonstrated manageable toxicity and substantial activity in patients with relapsed/refractory NKTCL previously treated with CPI, supporting further evaluation of nuclear export inhibition as a strategy to re-engage antitumor immunity after failure of PD-1 blockade.

**ClinicalTrials.gov identifier:**

NCT04425070.

## Graphical abstract

**Graphical Abstract:**
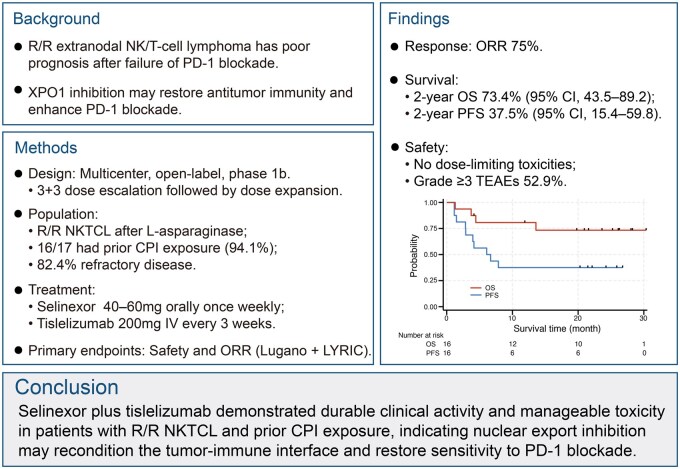

Implications for Practice Patients with R/R NKTCL whose disease progressed during or after PD-1 blockade–based immunotherapy have few established treatment options. This phase 1 b study suggests that combining selinexor, an oral XPO1 inhibitor, with tislelizumab, a PD-1 antibody, may offer a feasible treatment strategy for this difficult clinical setting. The regimen showed clinically meaningful responses, including in patients with disease refractory to prior CPI therapy, with manageable toxicity using a once-weekly selinexor schedule. These findings support further evaluation of selinexor plus PD-1 blockade as a potential post–PD-1 treatment approach in patients with R/R NKTCL.

## Introduction

Extranodal natural killer/T-cell lymphoma (NKTCL) is an aggressive, Epstein–Barr virus (EBV)–associated non-Hodgkin lymphoma that is more prevalent in East Asia and Latin America and typically involves extranodal sites such as the nasal cavity and upper aerodigestive tract.^1^ Outcomes remain poor once patients relapse or become refractory after first-line asparaginase-containing regimens. In a multicenter analysis, the median progression-free survival (PFS) after relapse was 4.1 months and overall survival (OS) was 6.4 months^2^; another cohort reported a median OS of 4.6 months following first relapse or progression.^3^ These observations highlight an urgent need for effective salvage strategies in relapsed/refractory (R/R) NKTCL.

PD-1/PD-L1 blockade has emerged as an active therapeutic class in NKTCL, consistent with EBV-driven immune evasion and frequent PD-L1 upregulation. Retrospective studies have reported variable response rates to PD-1 blockade (overall response rate [ORR] 40%-100%).^4–7^ Prospective data, however, have been heterogeneous. Avelumab monotherapy achieved an ORR of ∼38%,^8^ whereas the ORIENT-4 trial of sintilimab (*n* = 28) demonstrated an ORR of 75% with a 24-month OS of 78.6%.^9^ In the phase II GEMSTONE-201 study, sugemalimab monotherapy in R/R NKTCL achieved an ORR of 46.2% with durable responses and a manageable safety profile.^10^ Despite these advances, there remains no validated therapeutic strategy for patients who relapse after or are refractory to prior checkpoint inhibitor (CPI) therapy, a population characterized by particularly poor prognosis and limited treatment options.

Selinexor is a first-in-class selective inhibitor of nuclear export (SINE) that blocks exportin-1 (XPO1)-mediated transport of tumor suppressor proteins and oncogenic drivers,^11^ thereby restoring nuclear retention and promoting anti-tumor activity and apoptosis.^12^ Beyond direct anti-lymphoma effects, XPO1 inhibition provides a mechanistic rationale for combination with immune checkpoint blockade. In preclinical tumor models, selinexor increased checkpoint-related gene expression in immune and tumor cells and enhanced antitumor activity when combined with anti-PD-1 or anti-PD-L1 antibodies, accompanied by immune remodeling including increased natural killer cells and Th1-polarized CD4^+^ T cells.^13^ Although these data were generated in solid tumor models rather than ENKTL and were not specific to tislelizumab, they provide class-level nonclinical support for combining XPO1 inhibition with PD-1/PD-L1 blockade. XPO1 inhibition may also modulate EBV-related biology, providing an additional rationale in EBV-driven NKTCL.^13^^,^^14^ Clinically, a retrospective case series (*n* = 5) reported that selinexor combined with the PD-1 antibody tislelizumab induced durable responses even in patients with central nervous system involvement and disease progression after prior CPI therapy.^15^

On the basis of this mechanistic and clinical rationale, we conducted the TOUCH phase 1b trial to evaluate selinexor plus tislelizumab in patients with R/R NKTCL. Here, we report the results of Arm C, with particular emphasis on activity among patients with prior CPI exposure and treatment tolerability.

## Methods

### Study design and treatment

This open-label, multicenter, single-arm phase 1b study evaluated the safety, tolerability, and preliminary efficacy of selinexor plus tislelizumab in patients with R/R NKTCL. The trial comprised a dose-escalation phase followed by a dose-expansion phase. In dose escalation, a standard 3 + 3 design was used to identify dose-limiting toxicities (DLTs) and the maximum tolerated dose (MTD) (definitions in the Supplementary Methods). If neither DLTs nor an MTD were reached, the Safety Review Committee selected a recommended dose for expansion to further assess tolerability and preliminary antitumor activity in patients previously treated with L-asparaginase–containing regimens and CPI therapy.

Selinexor was administered orally at 40 mg once weekly, with escalation to 60 mg once weekly on days 1, 8, and 15 of each 21-day cycle. Tislelizumab was administered intravenously at a fixed dose of 200 mg every 3 weeks (day 1 of each cycle). The selinexor dose levels were prespecified before study initiation based on prior clinical experience with selinexor plus PD-1 blockade in NKTCL and the goal of maintaining long-term tolerability in combination therapy. Because objective responses had been observed with weekly selinexor doses of 40-60 mg in our prior NKTCL series,^15^ 60 mg once weekly was defined in the protocol as the highest planned dose level. Treatment continued until disease progression, unacceptable toxicity, or withdrawal of consent. Patients with indeterminate response (IR) per LYRIC could continue study treatment at the investigator’s discretion if clinically stable, with repeat imaging until PD was confirmed.

Eligible patients were aged ≥18 years with histologically confirmed R/R NKTCL according to the WHO 2016 classification and prior exposure to ≥1 L-asparaginase–containing regimen. Relapsed disease was defined as complete response (CR) after the most recent systemic therapy followed by documented relapse >6 months after treatment completion; refractory disease was defined as failure to achieve CR, or relapse ≤6 months after achieving CR. Additional eligibility criteria included an Eastern Cooperative Oncology Group (ECOG) performance status of 0-2, life expectancy ≥3 months, and ≥1 measurable lesion per Lugano 2014 criteria. Key exclusion criteria included another malignancy within 2 years, active autoimmune disease, or hemophagocytic syndrome. The full eligibility criteria are provided in the Supplementary Methods.

The study was approved by the institutional review board at each participating site and conducted in accordance with the Declaration of Helsinki and Good Clinical Practice guidelines. All patients provided written informed consent.

### Endpoints and assessment

The co-primary endpoints were the incidence of adverse events (AEs) and serious adverse events, graded according to the National Cancer Institute Common Terminology Criteria for Adverse Events version 5.0, and ORR, defined as the proportion of patients achieving CR or partial response (PR) per Lugano 2014 response criteria incorporating the LYRIC immune-response modification for immunotherapy-treated patients.^16^ Secondary endpoints included PFS, duration of response (DOR), and OS.

At baseline, patients underwent 18F-fluorodeoxyglucose positron emission tomography/computed tomography (18F-FDG PET/CT) and contrast-enhanced CT. Tumor assessments were performed every 2 cycles through cycle 6 and then every 3 cycles thereafter until radiographic progressive disease, unacceptable toxicity, or withdrawal of consent.

### Statistical analysis

The planned sample size was 16-28 patients with prior CPI exposure, including 6-12 patients in dose escalation to characterize tolerability and to inform dose selection for expansion. The safety analysis set included all patients who received at least 1 dose of study treatment. The efficacy analysis set included patients with prior CPI exposure (target *n* ≥ 16).

Baseline characteristics were summarized using descriptive statistics (median [range] for continuous variables; counts and percentages for categorical variables). Time-to-event endpoints (PFS and OS) were estimated using the Kaplan–Meier method. For PFS, an event was defined as the first occurrence of IR (per LYRIC), radiographic PD, or death from any cause, whichever occurred first; patients without an event were censored at the date of last adequate disease assessment. All analyses were performed using STATA/MP version 18.0.

## Results

### Patients

Between February 2023 and December 2023, 17 patients were enrolled (selinexor 40 mg, *n* = 3; 60 mg, *n* = 14). The median age was 51 years (range, 20-80), and 9 patients (52.9%) were male. Most patients had an ECOG performance status of 1-2 (76.5%) and stage IV disease (76.5%). A PINK score ≥2 was observed in 64.7% of patients, and PINK-E ≥ 3 in 58.8%. Plasma EBV DNA was detectable at baseline in 52.9%. Patients had received a median of 2 prior lines of therapy (range, 1-5), and 47.1% had received ≥3 lines. Sixteen patients (94.1%) had prior CPI exposure, including prior tislelizumab in 47.1%. Disease status at study entry was refractory in 82.4% (Table 1). Detailed individual-level information on prior CPI-containing therapy, including CPI agent, regimen type, combination partner(s), best response, treatment duration, reason for discontinuation, and washout interval before enrollment, is provided in Table S1.

**Table 1:** Baseline characteristics of patients.

Patient characteristics (*n* = 17)	Number (%)
**Median age (range), years**	51 (20-80)
**Gender**	
** Male**	9 (52.9)
** Female**	8 (47.1)
**ECOG**	
** 0**	4 (23.5)
** 1-2**	13 (76.5)
**Lugano staging**	
** I-II**	4 (23.5)
** IV**	13 (76.5)
**PINK score**	
** 0**	3 (17.6)
** 1**	3 (17.6)
** ≥2**	11 (64.7)
**PINK-E score**	
** 0-1**	5 (29.4)
** 2**	2 (11.8)
** ≥3**	10 (58.8)
**Positive plasma EBV-DNA**	9 (52.9)
**Prior treatment**	
** Median lines of treatment**	2 (1-5)
** ≥3 lines**	8 (47.1)
** Prior CPI exposure**	16 (94.1)
** Tislelizumab-exposed**	8 (47.1)
**Disease status before enrollment**	
** Relapsed**	3 (17.6)
** Refractory**	14 (82.4)

Data are shown as median (range) or *n* (%), as appropriate. Plasma EBV-DNA positivity was defined according to institutional quantitative PCR assay at baseline; EBV-DNA negativity was defined as a viral load <500 copies/mL.

Abbreviations: CPI, immune checkpoint inhibitor; ECOG, Eastern Cooperative Oncology Group performance status; PINK, Prognostic Index of Natural Killer lymphoma; PINK-E, PINK with incorporation of Epstein–Barr virus (EBV) DNA status.

### Safety

No DLTs were observed and the MTD was not reached. Overall, grade ≥3 treatment-emergent adverse events (TEAEs) occurred in 52.9% of patients (9/17), including 66.7% (2/3) at 40 mg and 50.0% (7/14) at 60 mg. The most frequent TEAEs (any grade, ≥20%) were anemia (82.4%), neutropenia (82.4%; grade ≥3, 29.4%), asthenia (76.5%), nausea (64.7%), vomiting (58.8%), decreased appetite (52.9%), weight loss (52.9%), thrombocytopenia (47.1%; grade ≥3, 11.8%), lymphocytopenia (47.1%; grade ≥3, 23.5%), proteinuria (41.2%), pneumonia (29.4%), hypothyroidism (29.4%), and alanine/aspartate aminotransferase elevations (23.5% each; grade ≥3, 5.9%). Serious treatment-related adverse events occurred in 23.5%. Detailed adverse event profiles are provided in Table 2.

**Table 2:** Summary of TEAEs.

Response	Sel 40 mg (*n* = 3)	Sel 60 mg (*n* = 14)	All patients (*n* = 17)
**TEAEs (any grade ≥3) *n* (%)**	2 (66.7)	7 (50.0)	9 (52.9)
**TEAEs (≥20% incidence)** **(any grade/≥grade 3)**			
** Anemia**	1 (33.3)/0	12 (85.7)/0	14 (82.4)/0
** Neutropenia**	2 (66.7)/0	12 (85.7)/5 (35.7)	14 (82.4)/5 (29.4)
** Thrombocytopenia**	0/0	8 (57.1)/2 (14.3)	8 (47.1)/2 (11.8)
** Lymphocytopenia**	1 (33.3)/1 (33.3)	7 (50.0)/3 (21.4)	8 (47.1)/4 (23.5)
** Asthenia**	2 (66.7)/0 (0)	11 (78.6)/0	13 (76.5)/0
** Nausea**	1 (33.3)/0	10 (71.4)/0	11 (64.7)/0
** Vomit**	1 (33.3)/0	9 (64.3)/0	10 (58.8)/0
** Decreased appetite**	1 (33.3)/0	8 (57.1)/0	9 (52.9)/0
** Weight loss**	2 (66.7)/0	7 (50.0)/0	9 (52.9)/0
** Proteinuria**	1 (33.3)/0	5 (35.7)/0	7 (41.2)/0
** Pneumonia**	1 (33.3)/0	4 (28.6)/0	5 (29.4)/0
** Hypothyroidism**	1 (33.3)/0	4 (28.6)/0	5 (29.4)/0
** ALT increased**	0/0	5 (35.7)/1 (7.1)	5 (29.4)/1 (5.9)
** AST increased**	0/0	4 (28.6)/1 (7.1)	4 (23.5)/1 (5.9)
** Fever**	1 (33.3)/0	3 (21.4)/0	4 (23.5)/0
** COVID-19**	1 (33.3)/0	3 (21.4)/0	4 (23.5)/0
** Dizziness**	1 (33.3)/0	3 (21.4)/0	4 (23.5)/0

Abbreviations: ALT, alanine aminotransferase; AST, aspartate aminotransferase; COVID-19, coronavirus disease 2019; TEAEs, treatment-emergent adverse events.

### Treatment exposure and tolerability

In the safety population (*n* = 17), selinexor plus tislelizumab was administered for a median of 8 cycles (range, 1-38), corresponding to a median treatment duration of 6.4 months (range, 1.1-26.7). The maximum treatment duration observed was 26.7 months. Treatment interruptions or delays occurred in 9 patients (52.9%), mainly due to thrombocytopenia or neutropenia. Selinexor dose reductions were required in 7 patients (41.2%), most commonly for thrombocytopenia, asthenia, or nausea. All interruptions/delays and dose reductions occurred at the 60-mg dose level (9/14 [64.3%] and 7/14 [50.0%], respectively). The regimen was managed with supportive care and dose modification; one patient discontinued treatment because of persistent anorexia and weight loss.

### Efficacy

**Figure 1.**
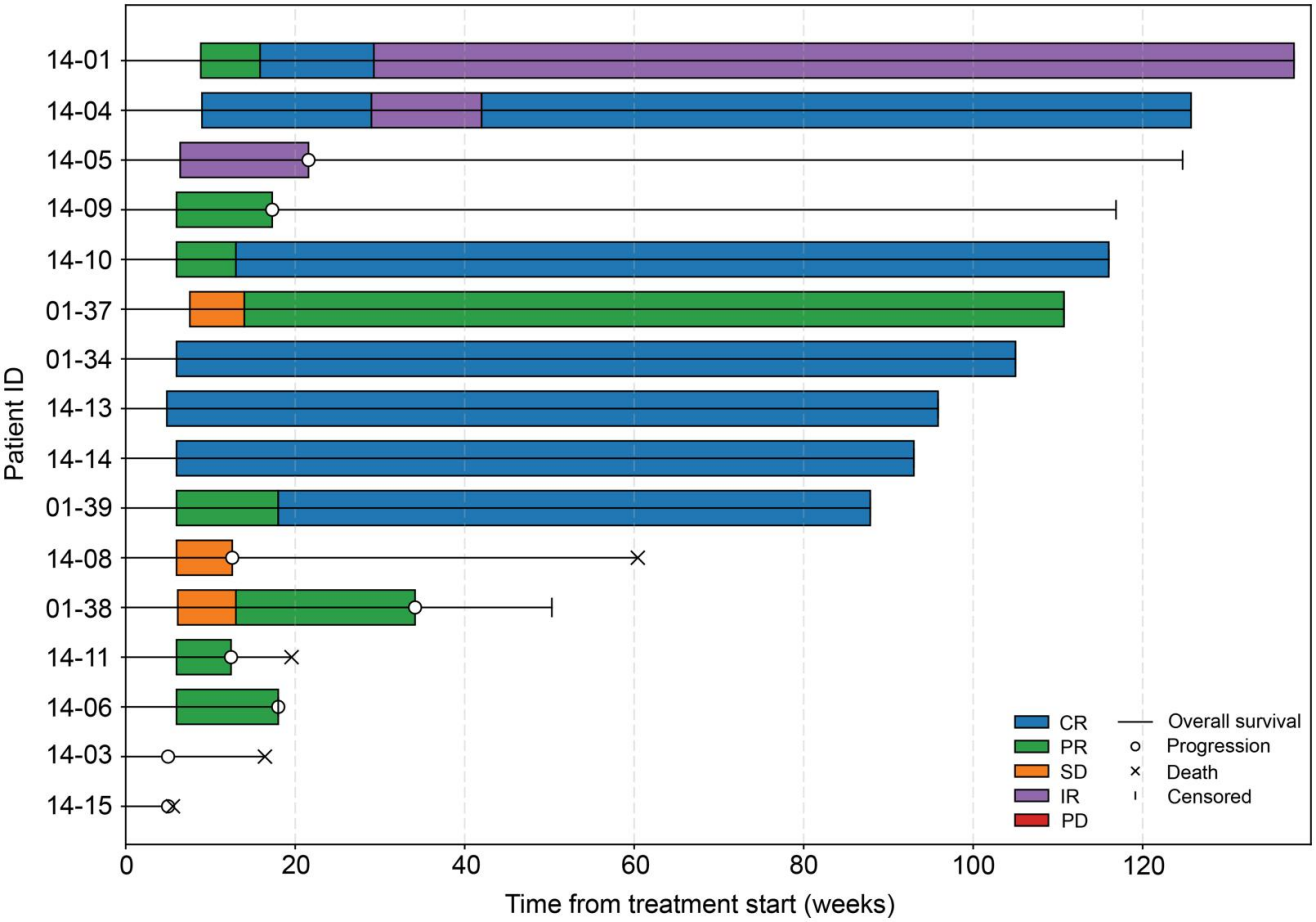
Treatment exposure and response dynamics. Swimmer plot of treatment exposure and response in patients with prior CPI exposure (*n* = 16). Bars represent overall survival from treatment initiation to death or last follow-up, ordered by duration. Colors indicate on-treatment response per Lugano 2014 with LYRIC modification (CR, PR, IR, SD, PD). Immune-related (IR) progression was counted as a PFS event but did not mandate treatment discontinuation. Sequential changes in response status are shown.

The efficacy analysis set included 16 patients with prior CPI exposure. One patient without prior CPI exposure withdrew for personal reasons was excluded. Among patients with prior CPI exposure, the ORR was 75.0% (12/16), including CR in 43.8% (7/16) and PR in 31.3% (5/16). In the subgroup with primary CPI-refractory disease (*n* = 6), the ORR was 50.0% (3/6), including CR in 33.3% (2/6) and PR in 16.7% (1/6). Response rates across additional prespecified subgroups are summarized in Table 3, whereas the swimmer plot (Figure 1) highlights the heterogeneity and dynamics of response over time, including immune-response patterns and response deepening from PR/IR to CR. Among the 12 patients who had CR or PR, the median DOR was 6.1 months (95% CI, 2.6–NE), with an observed range of 1.5-25.3 months; 6 of 12 responses were ongoing at the data cutoff.

**Table 3:** Best overall response to selinexor plus tislelizumab.

Response	Sel 40 mg (*n* = 3)	Sel 60 mg (*n* = 14)	CPI exposed (*n* = 16)	CPI primary refractory (*n* = 6)	All patients (*n* = 17)
**ORR, *n* (%)**	1 (33.3)	11 (78.6)	12 (75.0)	3 (50.0)	12 (70.6)
**CR**	1 (33.3)	6 (42.8)	7 (43.8)	2 (33.3)	7 (41.2)
**PR**	0 (0.0)	5 (35.7)	5 (31.3)	1 (16.7)	5 (28.4)
**SD**	1 (33.3)	0 (0.0)	0 (0.0)	0 (0.0)	1 (5.9)
**PD**	1 (33.3)	2 (14.3)	4 (25.0)	3 (50.0)	4 (23.5)
**NE**	0 (0.0)	1 (7.1)	0 (0.0)	0 (0.0)	1 (5.9)

Abbreviations: CPI, checkpoint inhibitor; CR, complete response; IR, immune response; ORR, overall response rate; PD, progressive disease; PR, partial response; SD, stable disease.

### Survival

**Figure 2.**
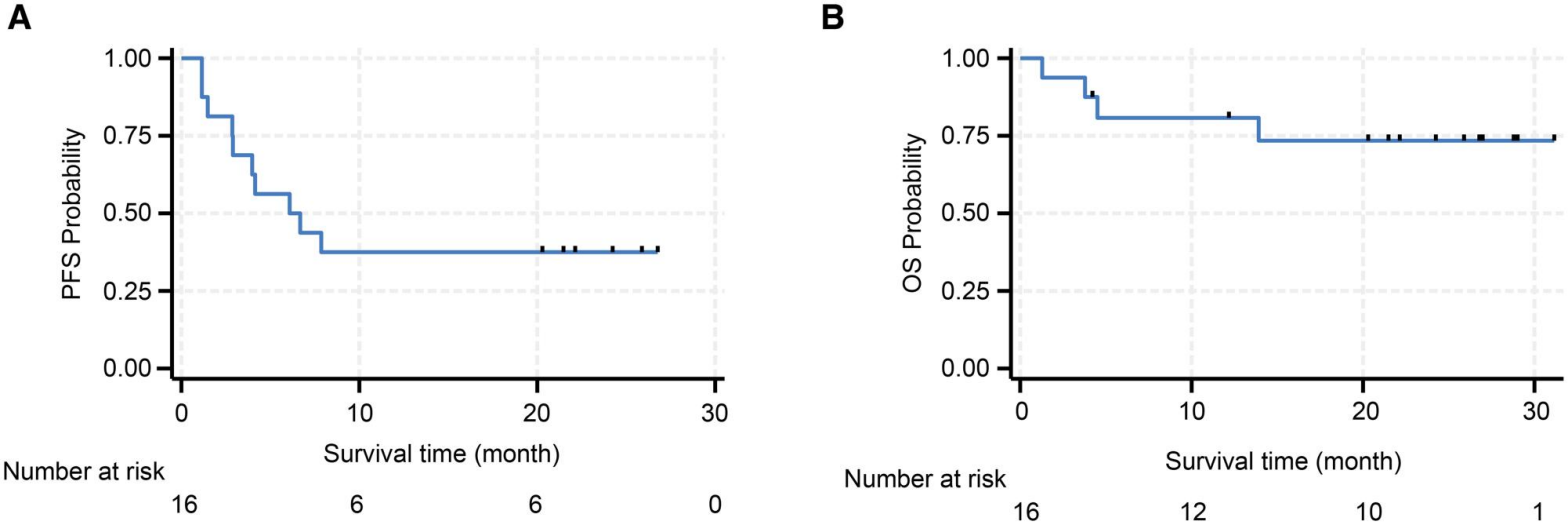
Progression-free survival and overall survival. Kaplan-Meier curves of (A) progression-free survival and (B) overall survival in patients with prior CPI exposure (*n* = 16). PFS events included immune-related progression per Lugano 2014 with LYRIC modification. Patients without events were censored at last follow-up.

At the data cutoff in September 2025, the median follow-up was 21.7 months (range, 1.3-31.1). Among the 16 patients included in the efficacy analysis, 10 had experienced disease progression and 4 had died; 1 patient was lost to follow-up. Five of the 10 patients with progression received subsequent PD-1 blockade–based therapy. The median PFS was 6.1 months (95% CI, 2.9-not estimable [NE]), with a 2-year PFS rate of 37.5% (95% CI, 15.4-59.8) (Figure 2A). The median OS was not reached (95% CI, 13.9–NE), and the 2-year OS rate was 73.4% (95% CI, 43.5-89.2; Figure 2B). The durability and evolution of responses over time are illustrated in the swimmer plot (Figure 1).

## Discussion

In this phase 1 b study, selinexor combined with tislelizumab demonstrated substantial and durable activity in patients with R/R NKTCL who had all previously received PD-1 blockade. Among 16 patients with prior CPI exposure, the ORR was 75.0%, including 43.8% CRs, and the ORR was 50.0% among patients with primary CPI-refractory disease. With a median follow-up of 21.7 months, the 2-year OS rate reached 73.4%, and median OS was not reached. These outcomes compare favorably with historical cohorts in which patients whose disease progressed after asparaginase-based therapy, particularly after immunotherapy, had a median OS of approximately 4-6 months.^2^^,^^3^ Although cross-study comparisons must be interpreted cautiously, the magnitude and durability of responses observed here suggest that PD-1 resistance in NKTCL may not represent an irreversible immune escape state.

The activity observed is unlikely to be attributable to either agent alone. PD-1 retreatment after progression has not produced consistent durable benefit in R/R NKTCL.^6^^,^^8^^,^^17^^,^^18^ In broader hematologic malignancies, single-agent or selinexor-based activity has been reported mainly with higher-intensity dosing schedules than the once-weekly 40-60 mg schedule used here. In contrast, selinexor plus tislelizumab induced objective responses in a cohort enriched for prior CPI exposure and resistance, including in primary CPI-refractory disease. Among patients who had CR or PR, the median duration of response was 6.1 months (95% CI, 2.6–NE), with 6 of 12 responses ongoing at data cutoff, and treatment exposure was sustained (median, 8 completed 21-day cycles; maximum, 26.7 months). The delayed and evolving response patterns observed under LYRIC are inconsistent with simple cytotoxic effects and instead indicate immune re-engagement facilitated by XPO1 inhibition in the context of PD-1 blockade. Although the single-arm design cannot formally isolate the contribution of each agent, the dose intensity, response magnitude, and activity in primary CPI-refractory disease collectively support a clinically relevant combination effect.

The concept that immune resistance in NKTCL may be modifiable is supported by independent clinical evidence. In the SCENT trial, the combination of sintilimab and the histone deacetylase inhibitor chidamide achieved an ORR of 58% and CR rate of 42%, including responses in patients previously exposed to checkpoint inhibitors.^19^ Although mechanistically distinct, both nuclear export inhibition and epigenetic modulation converge on altering transcriptional programs and immune signaling pathways. These findings collectively suggest that PD-1 resistance in EBV-driven NKTCL is not a fixed endpoint but may reflect reversible immune dysfunction.

Mechanistically, XPO1 inhibition provides a plausible framework for such immune reprogramming. XPO1 regulates nuclear export of tumor suppressors and transcription factors critical for immune signaling. Pharmacologic blockade enhances type I interferon signaling, augments antigen presentation, and modulates T-cell and myeloid cell function within the tumor microenvironment.^13^^,^^20–22^ In preclinical tumor models, selinexor combined with PD-1/PD-L1 blockade produced greater antitumor activity than either approach alone, accompanied by immune remodeling.^13^ Impaired interferon signaling and defective antigen presentation have been implicated in resistance to checkpoint blockade across malignancies; restoration of these pathways may re-enable effective immune recognition. In virus-associated cancers, XPO1 inhibition also suppresses herpesvirus lytic replication and strengthens innate antiviral responses.^23^^,^^24^ Given the central role of EBV in NKTCL pathogenesis, simultaneous modulation of viral transcription and host immunity may be particularly relevant. The convergence of these immunologic and antiviral effects supports a mechanistic rationale for the observed clinical activity.

The tolerability profile further strengthens the therapeutic rationale. Selinexor was administered at 40-60 mg once weekly—substantially lower than regimens used in multiple myeloma (STORM: 80 mg twice weekly) or diffuse large B-cell lymphoma (SADAL: 60 mg twice weekly). In those trials, grade ≥3 thrombocytopenia occurred in 59% and 46% of patients, respectively, with treatment discontinuation rates of 17%-18%.^25^^,^^26^ In contrast, grade ≥3 thrombocytopenia and neutropenia in the present study occurred in 12% and 29%, respectively, and no patient discontinued therapy due to hematologic toxicity. Any-grade anemia and neutropenia were common, but severe anemia was not observed and neutropenia was manageable with supportive care and dose modification. The lower incidence of severe thrombocytopenia may reflect both the once-weekly lower-dose schedule and differences in prior treatment exposure, as patients with NKTCL may have had less cumulative exposure to strongly myelosuppressive cytotoxic therapy than heavily pretreated DLBCL or multiple myeloma populations. Although dose interruptions and reductions were frequent at 60 mg, events were manageable with supportive care. Notably, meaningful and durable responses were observed despite reduced dose intensity. This observation suggests that the efficacy of the combination may derive from immunologic synergy rather than maximal cytotoxic exposure, raising the possibility that lower, immunomodulatory dosing of XPO1 inhibition may be sufficient when paired with checkpoint blockade.

This study has limitations, including its single-arm design and small sample size. Post-progression PD-1 blockade–based therapy in some patients may confound OS interpretation. Nevertheless, the consistency of responses among patients with prior CPI exposure and those with CPI-refractory disease, the durability of disease control, and the mechanistic plausibility collectively provide a coherent signal. While definitive conclusions require validation in larger cohorts, these findings support the hypothesis that nuclear export inhibition can recondition the tumor–immune interface and restore sensitivity to PD-1 blockade in NKTCL.

Future investigations should incorporate prospective biomarker analyses to refine patient selection. Longitudinal EBV-DNA kinetics,^27^ PD-L1 expression patterns,^28–30^ and interferon or antigen-presentation signatures may help define biological subsets most likely to benefit. More broadly, redefining PD-1 resistance as a potentially reversible state has implications beyond NKTCL and may inform rational immunotherapy combinations in other virus-associated or immune-evasive malignancies.

## Supplementary Material

oyag266_Supplementary_Data

## Data Availability

The deidentified individual participant data that support the findings of our study are available from the corresponding author upon reasonable request (correspondence to Dr Rong Tao: rtao@shca.org.cn).
