# Supplementary material for: Selinexor plus tislelizumab in patients with relapsed/refractory natural killer/T-cell lymphoma after failure of PD-1 blockade: the phase 1b TOUCH trial
Source: Oncologist. 2026 Jul 11;31(9):oyag266. doi: 10.1093/oncolo/oyag266 (PMC13451085; doi:10.1093/oncolo/oyag266)
Supplement: Supplementary file 1 [file oyag266_supplementary_data.zip › Supplementary Eligibility.pdf]

Eligibility

Inclusion Criteria

Subjects must meet **all** of the following criteria to be eligible for enrollment:

1. **Able and willing** to provide written informed consent (ICF).
2. Age **≥18 years**.
3. Histologically confirmed **relapsed/refractory (R/R) mature T-cell and NK-cell lymphoma**, classified according to the **2016 WHO Classification of Tumours of Haematopoietic and Lymphoid Tissues**.
4. Prior systemic therapy:
   - For **mature T-cell lymphoma**: previously received **≥1 systemic treatment regimen**, including agents such as **anthracyclines**.
   - For **extranodal NK/T-cell lymphoma (ENKTL)**: must have received **systemic therapy containing L-asparaginase**.
   - **Radiotherapy alone** as prior therapy for mature T-cell or NK-cell lymphoma is **not acceptable**.
5. R/R disease after the **most recent** treatment:
   - **Relapse** is defined as achieving **CR** after the most recent systemic therapy, with **documented disease recurrence >6 months** after completion of that therapy.
   - **Refractory** is defined as **failure to achieve CR** after the most recent systemic therapy, **or** achieving CR with a **duration of CR ≤6 months**.
6. Measurable/evaluable disease per the **revised lymphoma response criteria**:
   - **Measurable disease** is defined as **at least one lesion** that can be clearly measured in **two perpendicular dimensions**.
   - For **nodal target lesions**, the minimum **longest diameter (LD)** must be **>1.5 cm**.
   - For **extranodal lesions**, the LD must be **>1.0 cm**.
   - For **ENKTL**, the presence of **evaluable lesions confirmed by PET-CT** meets eligibility; **measurable disease is not mandatory**.
7. **ECOG performance status** of **0 or 1**.

8. Clinical chemistry meeting all of the following:
   a. **AST** and **ALT ≤2.5 × ULN**.
   b. **Total bilirubin ≤1.5 × ULN** (except subjects with confirmed **Gilbert's syndrome**, permitted up to **3 × ULN**).
   c. **Creatinine clearance ≥50 mL/min**, estimated by the **Cockcroft–Gault** formula or measured by **24-hour urine collection**.
9. Adequate bone marrow reserve, defined as:
   a. **ANC ≥1.0 × 10^9/L**, with **no granulocyte colony-stimulating factor** administered within **7 days** prior to testing.
   b. **Platelets ≥75 × 10^9/L**; for subjects with **lymphoma bone marrow involvement**, platelets **≥50 × 10^9/L**, with **no platelet transfusion and/or thrombopoietic growth factors** administered within **7 days** prior to testing.
   c. **Hemoglobin ≥80 g/L**.
10. Coagulation: **INR** and **PT ≤1.5 × ULN**.
11. Except for **hearing loss, alopecia, and hyperpigmentation**, all toxicities from prior anti-tumor therapy have recovered to **≤ Grade 1** per **NCI-CTCAE v5.0**.
12. Life expectancy >**3 months**.
13. Women of childbearing potential must meet **both** of the following:
   a. Agree, from the date of signing the ICF, to use **two investigator-approved contraceptive methods simultaneously** or practice **complete abstinence** during study drug use and for **3 months after** the last dose of study drug.
   b. Have a **negative serum pregnancy test** at screening.
14. Male subjects (including those with prior vasectomy) must agree to use a **condom** during sexual activity with women of childbearing potential and, from the date of signing the ICF, have **no plan to impregnate** a partner during study drug use and for **3 months after** the last dose of study drug.
15. Thyroid function: **TSH** and **free T3/free T4** within the normal reference range (**Cohort C only**).
16. The proportion of subjects who previously received **anti-PD-1 antibody therapy** and **never achieved PR or better** must be **≤25%** (**Cohort C only**).

4.4 Exclusion Criteria

Subjects will **not** be enrolled if any of the following apply:

1. **Active hepatitis B or hepatitis C infection**:
   a. **HBsAg positive** or **HBcAb positive**. For subjects who are **HBsAg negative and HBcAb positive**, enrollment is allowed if **HBV DNA is undetectable**. The upper limit of normal for HBV DNA is per each site's assay.
   b. **HCV antibody positive**. For subjects who are HCV antibody positive, enrollment is allowed if **HCV RNA is undetectable**. The upper limit of normal for HCV RNA is per each site's assay.
2. Known history of **HIV infection** and/or **AIDS**.
3. **Major surgery** within **4 weeks** prior to first dose of study drug.
4. Any other anti-cancer therapy within **3 weeks** prior to first dose of study drug, including **radiotherapy, chemotherapy, immunotherapy, hormonal therapy, biologic therapy, traditional Chinese medicines/chinese patent medicines with anti-tumor effects, investigational drugs**, etc.
5. Prior **allogeneic hematopoietic stem cell transplantation (allo-HSCT)**.
6. Receipt of any **anti-infective vaccine** (e.g., influenza vaccine, varicella vaccine) within **4 weeks** prior to first dose of study drug.
7. Prior exposure to a **selective inhibitor of nuclear export (SINE)**, including **ATG-010**.
8. History of **stroke** or **intracranial hemorrhage** within **6 months** prior to first dose, or **active Grade ≥3 gastrointestinal bleeding**.
9. Clinically significant cardiovascular disease, including:
   a. **Myocardial infarction** within **6 months** prior to screening.
   b. **Unstable angina** within **3 months** prior to screening.
   c. Clinically significant arrhythmia (e.g., sustained ventricular tachycardia, ventricular fibrillation, torsades de pointes).
   d. **Mobitz type II second-degree** or **third-degree** atrioventricular block.

e. **NYHA Class III or IV** congestive heart failure.

f. Echocardiographic **LVEF <50%**.

10. Inability to swallow tablets; **malabsorption syndrome**; or any gastrointestinal disease/dysfunction that may interfere with **ATG-010 absorption**.
11. Active infection requiring **parenteral antibiotics, antivirals, or antifungals** within **1 week** prior to first dose (prophylactic anti-infective use is allowed).
12. Known hypersensitivity to **ATG-010**, or any drugs in the **ICE/GEMOX** regimens, or **anti-PD-1 antibody**, or any excipients.
13. **Pregnant or breastfeeding** women.
14. In the investigator's judgment, comorbidities or other conditions that may affect protocol compliance or make the subject unsuitable for participation.

Additional exclusion criteria for Cohort C (in addition to the above)

18. CNS involvement:

- **Phase I**: subjects with **CNS involvement** are excluded.
- **Phase II**: subjects **with or without symptoms** of CNS involvement may be considered for enrollment, but must have **imaging-evaluable lesions**.

19. Concomitant **hemophagocytic syndrome (HPS/HLH)**.
20. **Adult T-cell leukemia/lymphoma (ATLL)**.
21. Active autoimmune disease or a history of autoimmune disease with potential recurrence (see p.104). **Note:** subjects with the following conditions may be further screened:
    a. Controlled **type 1 diabetes mellitus**.
    b. **Hypothyroidism** on hormone replacement only.
    c. Controlled **celiac disease**.
    d. Skin disorders not requiring systemic therapy (e.g., vitiligo, psoriasis, alopecia).
    e. Any other disease not expected to recur without an external trigger.
22. Any condition requiring systemic treatment with **corticosteroids (>10 mg/day prednisone or equivalent)** or other immunosuppressive drugs within **14 days** prior to first dose. Exceptions include subjects currently or previously receiving:

a. **Adrenal replacement** steroids (≤10 mg/day prednisone or equivalent).

b. Topical, intraocular, intra-articular, intranasal, or inhaled corticosteroids with minimal systemic absorption.

c. Short-term (≤7 days) prednisone for prophylaxis (e.g., contrast allergy) or treatment of non-autoimmune conditions (e.g., delayed hypersensitivity due to allergen exposure).

23. Known history of **interstitial lung disease**, **non-infectious pneumonitis**, **pulmonary fibrosis**, or **acute lung disease**.
24. **Autologous stem cell transplantation (auto-HSCT)** within **6 months** prior to first dose.
25. Another primary malignancy occurring within **2 years** prior to first dose (except locally curable malignancies treated with curative intent).
26. Prior **anti-PD-1 or anti-PD-L1 monoclonal antibody** therapy with occurrence of **Grade ≥3 immune-related adverse events**.
